# Structure and function relationship of formate de­hydrogenases: an overview of recent progress

**DOI:** 10.1107/S2052252523006437

**Published:** 2023-09-01

**Authors:** Ami Kobayashi, Midori Taketa, Keisei Sowa, Kenji Kano, Yoshiki Higuchi, Hideaki Ogata

**Affiliations:** a Division of Applied Life Sciences, Graduate School of Agriculture, Kyoto University, Sakyo, Kyoto 606-8502, Japan; b Graduate School of Science, University of Hyogo, Koto 3-2-1 Kamigori, Ako, Hyogo 678-1297, Japan; c Office of Society Academia Collaboration for Innovation, Kyoto University, Gokasho, Uji 611-0011, Japan; University of Michigan, USA

**Keywords:** formate de­hydrogenases, biotechnological applications, *Methylorubrum extorquens* AM1, Mo/W enzymes

## Abstract

Formate de­hydrogenases (FDHs) catalyze the two-electron oxidation of formate to carbon dioxide. FDHs can be divided into several groups depending on their subunit composition and active-site metal ions. Metal-dependent (Mo- or W-containing) FDHs from prokaryotic organisms belong to the superfamily of molybdenum enzymes and are members of the di­methyl­sulfoxide reductase family. In this short review, recent progress in the structural analysis of FDHs together with their potential biotechnological applications are summarized.

## Abstract 2

Formate de­hydrogenases (FDHs) catalyze the two-electron oxidation of formate to carbon dioxide. This article presents recent progress in the structural analysis of FDHs together with their potential biotechnological applications.

## 1. Introduction

Formate de­hydrogenases (FDHs) catalyze the two-electron oxidation of formate to carbon dioxide and play an important role in several kingdoms of organisms (Meneghello, Léger & Fourmond, 2021 ▸; Calzadiaz-Ramirez & Meyer, 2022 ▸; Hartmann *et al.*, 2015 ▸; Nielsen *et al.*, 2019 ▸; Moon *et al.*, 2020 ▸; Maia *et al.*, 2017*b* ▸; Stripp *et al.*, 2022 ▸). Owing to its low redox potential [*E*°′ = −0.42 V; potentials are given with respect to the standard hydrogen electrode (SHE) in this review unless stated otherwise] (Calzadiaz-Ramirez & Meyer, 2022 ▸), formate is widely used by prokaryotes and eukaryotes to obtain energy via C1 metabolism with various physiological redox partners [nicotine adenine dinucleotide (NAD), cytochromes, ferredoxins, coenzyme F_420_ and quinones (Grimaldi *et al.*, 2013 ▸; Hartmann *et al.*, 2015 ▸; Hille *et al.*, 2014 ▸; Maia *et al.*, 2015 ▸, 2017*a* ▸)]. Because these biochemical metabolic mechanisms are essential across all organisms, numerous FDHs possessing different structures, subunit compositions and metabolic functions have been reported. In addition, the active sites of FDHs are highly diverse in terms of structure (*i.e.* metal ions, amino acids and coenzymes), catalytic turnover rate and O_2_ sensitivity. The conversion of formate to CO_2_ is the terminal enzymatic step in C1 unit oxidation to CO_2_ in *Methyl­orubrum extorquens* AM1. This α-proteobacterium possesses two pathways for the conversion of C1 units and utilizes multiple FDHs (Laukel *et al.*, 2003 ▸). The metal-containing formate de­hydrogenase 1 from *M. extorquens* AM1 (MeFDH1) comprises two subunits and is located in the cytoplasmic fraction. In this review, we provide a brief overview of the classification of FDHs and then discuss recent progress in the structural characterization of FDHs with a focus on MeFDH1, whose structure has recently been solved by X-ray crystallography and cryo-electron microscopy (cryo-EM).

## 2. Classification of formate de­hydrogenases

FDHs can be divided into two distinct classes according to their metal content. The first class of enzymes is metal-free NAD-dependent FDHs, which lack metallic active sites and catalyze the oxidation of formate by hydride transfer to NAD^+^ (Tishkov, 2004 ▸). Several NAD-dependent FDHs have been found in *Candida boidinii* (CbFDH), *Candida methyl­ica* (CmFDH), *Chaetomium thermophilum* (CtFDH), *Myceliophthora thermophila* (MtFDH) and *Thio­bacillus sp.* KNK65MA (TsFDH). They do not have metal ions or other redox centers and belong to the d-specific 2-hy­droxy­acid de­hydrogenase family (Vinals *et al.*, 1993 ▸). CbFDH is a well studied enzyme, probably because it is commercially available (Sultana *et al.*, 2016 ▸; Tishkov & Popov, 2006 ▸; Miyaji & Amao, 2020 ▸). Considering the redox potential of NAD^+^/NADH (*E*°′ = −0.32 V), NAD-dependent FDHs generally catalyze formate oxidation rather than CO_2_ reduction because the former is thermodynamically favorable. However, NAD-dependent MtFDH and CbFDH have also been reported to function as CO_2_ reductases (Miyaji & Amao, 2020 ▸; Altaş *et al.*, 2017 ▸).

The second class of enzymes is metal-dependent (Mo- or W-containing) FDHs from prokaryotic organisms, which belong to the superfamily of molybdenum enzymes and are members of the di­methyl­sulfoxide (DMSO) reductase family (Hille *et al.*, 2014 ▸). The active site contains a hexacoordinated metal ion (Mo or W) in a distorted triangular prismatic geometry. Two di­thiol­ene groups from two molybdopterin (MPT) moieties function as ligands. In addition, the coordination sphere of the metal ion is completed by a cysteine (Cys) or seleno­cysteine (SeCys) residue and an inorganic sulfide that is necessary for the activity (Thomé *et al.*, 2012 ▸; Arnoux *et al.*, 2015 ▸). As summarized in Table 1 ▸, Mo- or W-containing FDHs have been isolated and characterized from numerous prokaryotes. These prokaryotes include methyl­otrophs (*Methyl­orubrum extorquens* AM1, *Methyl­obacterium sp*. RXM and *Methyl­osinus thrichosporium*), methano­gens (*Methano­bacterium formicicum*, *Methano­coccus maripaludis* and *Methano­coccus vannielii*), sulfate-reducing bacteria (SRB; *Desulfovibrio alaskensis*, *Desulfovibrio desulfuricans*, *Desulfovibrio gigas*, *Desulfovibrio vulgaris* and *Syntrophobacter fumaroxidans*), acetogens (*Clostridium acidurici*, *Clostridium carboxidivorans*, *Clostridium formico­aceticum*, *Clostridium ljungdahlii*, *Clostridium pasteurianum*, *Clostridium thermoaceticum*, *Acetobacterium woodii* and *Thermoanaerobacter kuvui*), proteobacteria (*Rhodopseudomonas palustris*, *Rhodobacter aestuarii*, *Cupriavidus necator*, *Rhodobacter capsulatus*, *Escherichia coli* and *Pseudomonas aeruginosa*) *etc*. Although most FDHs are sensitive to oxygen, some are known to be active even under aerobic conditions. A number of reports have suggested that FDHs bearing a SeCys residue at the metallic active site appear to be rather sensitive to oxygen (Nielsen *et al.*, 2019 ▸, Bassegoda *et al.*, 2014 ▸). Metal-dependent FDHs are structurally very diverse. For example, *E. coli* can express one monomeric cytoplasmic enzyme (EcFDH-H) that contains only the Mo active center and one iron–sulfur ([Fe–S]) cluster and two heteromeric membrane-bound respiratory enzymes (EcFDH-N and O) that harbor seven additional redox partners including [Fe–S] clusters and heme moieties. Furthermore, some FDHs (CnFDH, MeFDH1, MtFDH, RcFDH and RpFDH) utilize flavin mononucleotide (FMN) as a catalytic site with NAD^+^ in a separate subunit to the one containing the metal (Mo or W) active site. The authors of a previous review proposed six categories of metal-dependent FDHs (Nielsen *et al.*, 2019 ▸) based on gene organization, subunit composition and cofactor requirements. Interestingly, certain FDHs (mostly containing W at the active site) have also been reported to act as CO_2_ reductases that are able to catalyze the reverse reaction.

## 3. Overall structures

**Figure 1:**
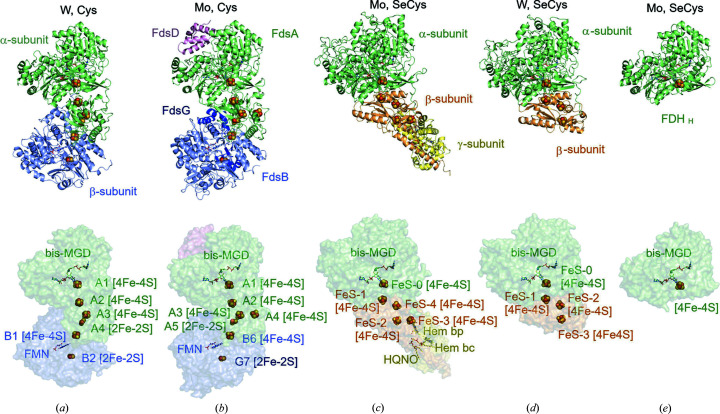
Structure comparison of FDHs from various organisms: (*a*) FDH from *Methyl­orubrum extorquens* AM1 (PDB entry 8j83; Kobayashi *et al.*, in the press), (*b*) FDH from *Rhodobacter capsulatus* (PDB entry 6tga; Radon *et al.*, 2020 ▸), (*c*) FDH-N from *Escherichia coli* (PDB entry 1kqf; Jormakka *et al.*, 2002 ▸), (*d*) FDH from *Desulfovibrio gigas* (PDB entry 1h0h; Raaijmakers *et al.*, 2002 ▸) and (*e*) FDH-H from *E. coli* (PDB entry 1fdo; Boyington *et al.*, 1997 ▸). The upper images show the protein and subunit structures, with the corresponding metal ion and ligand residue indicated above each image. The lower images show the arrangements of the cofactors, active sites and iron–sulfur clusters.

Metal-dependent FDHs are composed of several subunits, containing the W or Mo ion at the active site (see below) and the additional cofactors, *i.e.* [4Fe–4S] and [2Fe–2S] clusters, FMN/FAD, or hemes. Their subunit compositions vary greatly (Fig. 1 ▸). Four structurally distinct types of active sites can be identified on the basis of the metal ions (W or Mo) and coordinated amino acid residues (Cys or SeCys): (1) Mo–Cys type [*e.g.* FDH from *R. capsulatus* (RcFdsGBACD)], (2) Mo–SeCys type [*e.g.* FDH from *E. coli* (EcFDH-F)], (3) W–Cys type [*e.g.* FDH from *M. extorquens* (MeFDH1)] and (4) W–SeCys type [*e.g.* FDHs from *D. gigas* (DgFDH) and *D. vulgaris* (DvFDH-AB)]. FDHs containing all four types of active sites have now been characterized by crystallography or cryo-EM (Stripp *et al.*, 2022 ▸). In all cases, the α subunit, containing the Mo or W ions in the active site, displayed an approximate molecular mass of 85–100 kDa with a highly conserved overall structure and at least one [4Fe–4S] cluster proximal to the active site. In the electron transfer chain, the proximal [4Fe–4S] cluster is further connected to an additional Fe–S clusters in either the same α subunit or a closely located neighboring subunit.

Recently, we successfully determined the structure of MeFDH1, a W–Cys-type enzyme, by means of cryo-EM (Yoshikawa *et al.*, 2022 ▸) and X-ray crystallography. MeFDH1 consists of two subunits denoted α and β [Fig. 1 ▸(*a*)]. The α subunit contains the W–Cys active site with the proximal [4Fe–4S] cluster, along with an additional two [4Fe–4S] clusters and one [2Fe–2S] cluster. The β subunit harbors the binding site for NAD^+^ and possesses one FMN cofactor, one [4Fe–4S] cluster and one [2Fe–2S] cluster. The [4Fe–4S] cluster in the β subunit is connected to the [4Fe–4S] and [2Fe–2S] clusters in the α subunit with distances of 14.9 and 10.9 Å, respectively. The β subunit is responsible for catalyzing the NAD^+^/NADH redox couple in MeFDH1 and is therefore called the diaphorase unit.

Comparison of the α subunit of W–Cys-type MeFDH1 and some W–SeCys/Mo–SeCys-type FDHs showed that the overall structural architectures, including the locations of the active sites and proximal [4Fe–4S] clusters, are similar [Figs. 1 ▸(*c*)–1(*e*)]. The structure of W–Cys-type MeFDH1 is also very similar to that of Mo–Cys-type RcFdsGBACD [RcFDH homologously expressed in *R. capsulatus* with operon fdsGBACD (Radon *et al.*, 2020 ▸)], although the orientation of the β subunit with respect to the α subunit is slightly different. Note that the structures of the FdsA, FdsB and FdsG subunits of RcFdsGBACD are also similar to those of Nqo3, Nqo1 and Nqo2, respectively, of respiratory complex I from *Thermus thermophilus* (Tt complex I) (Baradaran *et al.*, 2013 ▸), as described below.

**Figure 2:**
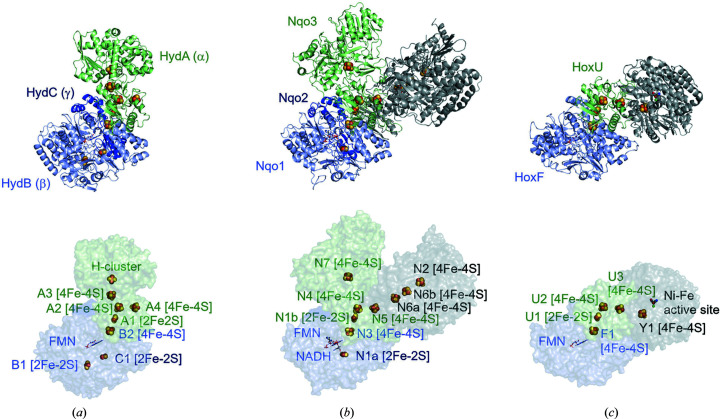
Comparison of structurally similar proteins: (*a*) electron-bifurcating [FeFe] hydrogenase from *Thermotoga maritima* (PDB entry 7p5h; Furlan *et al.*, 2022 ▸), (*b*) respiratory complex I from *Thermus thermophilus* (PDB entry 3iam; Berrisford & Sazanov, 2009 ▸) and (*c*) NAD^+^-reducing [NiFe] hydrogenase from *Hydrogeno­philus thermoluteolus* (PDB entry 5xf9; Shomura *et al.*, 2017 ▸). The upper images show the protein and subunit structures. The lower images show the arrangements of the cofactors, active sites and iron–sulfur clusters.

When FDHs are compared with other oxidoreductases (*e.g.* Tt complex I or hydrogenases), some interesting similarities are found in terms of not only the overall structures and relative orientations of the constituent subunits but also the spatial arrangements of the cofactors. As shown in Figs. 1 ▸(*a*) and 2 ▸(*b*), the overall structure of MeFDH1 is very similar to that of the Nqo1–2–3 subcomplex of Tt complex I. The Nqo3 subunit of Tt complex I does not possess the W metal center found in the α subunit of MeFDH1, but the N7 ([4Fe–4S]) cluster is located at the corresponding position, which is isolated from the electron transfer chain, although the W metal center is connected to the electron transfer chain by the proximal (A1) and next (A2) [4Fe–4S] clusters in MeFDH1. The relative arrangement of FMN and the other Fe–S clusters (N4, N1b, N3 and N1a) in the electron transfer chain of the diaphorase unit of Tt complex I is considerably similar to that of FMN and the clusters A3, A4, B1 and B2, respectively, in MeFDH1 [Figs. 1 ▸(*a*) and 2 ▸(*b*)]. In addition, Tt complex I has another electron transfer route connected to the cofactor chain composed of the clusters N5 (Nqo3), N6a-N6b (Nqo9) and N2 (Nqo6) in the subcomplex, which diverges at the N4 cluster from the electron transfer chain in the β subunit (diaphorase). Furthermore, the β subunit of MeFDH1 also displays structural similarity to the HoxFU subcomplex (diaphorase unit) in the NAD^+^-reducing [NiFe] hydrogenase (HtHoxFUHY) from *Hydrogeno­philus thermoluteolus* [Figs. 1 ▸(*a*) and 2 ▸(*c*)] (Shomura *et al.*, 2017 ▸). However, the surface region of the diaphorase unit (HoxFU), on which the hydrogenase subcomplex (HoxHY) makes a connection, is not the corresponding region of the β subunit of MeFDH1, but that of the Nqo1–2 subcomplex (diaphorase unit) in Tt complex I, and the electron transfer chain in HoxFU is connected to the clusters in the catalytic unit of HoxHY as found for those in the Nqo3–6–9 subcomplex of Tt complex I. Interestingly, cryo-EM studies of the electron-bifurcating [FeFe] hydrogenase from *Thermotoga maritima* (TmHyd­ABC) [Fig. 2 ▸(*a*)] revealed that its structure is not closely related to that of the [NiFe] hydrogenase HtHoxFUHY but more similar to that of MeFDH1, although the protomer of TmHydABC is composed of three subunits, α, β and γ (Furlan *et al.*, 2022 ▸). The diaphorase unit of TmHydABC is separated into two subunits, β and γ, which are merged into one subunit (β) in MeFDH1. The separation, fusion and/or partial insertion/deletion of protein subunits are often seen in the structures of oxidoreductases (*e.g.* the electron transfer subunit of [NiFe] hydrogenases is separated into HoxY and part of HoxU in the structure of TtHoxFUHY). In this context, from the viewpoint of the evolution of the subunit structures, it is interesting to clarify the function and/or the evolutionary reason for the existence of the N7 [4Fe–4S] cluster in Nqo3 of Tt complex I, which is isolated from the electron transfer chain. The active center (H-cluster) of TmHydABC occupies the same position as the W metal center (with the A1 cluster) of MeFDH1, and the relative arrangement of the clusters from the diaphorase unit (β and γ) to the catalytic unit (α) in the electron transfer chain is surprisingly conserved between them, although TmHydABC catalyzes the oxidoreduction of H_2_ as in the case of [NiFe] hydrogenases [Fig. 2 ▸(*a*)].

The structural features of both the subunit and cluster arrangements of these oxidoreductases strongly support the hypothesis that the subcomplexes have independently evolved and are assembled as prebuilt modules into the energy metabolism machinery in each case (Efremov & Sazanov, 2012 ▸; Shomura *et al.*, 2017 ▸).

## 4. Active site structures

**Figure 3:**
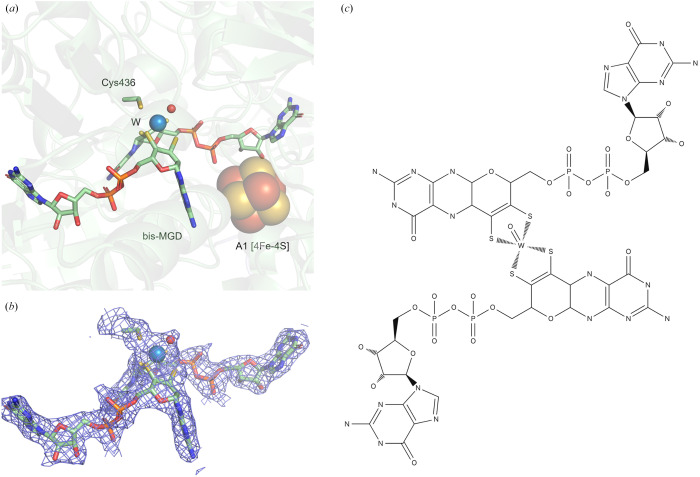
(*a*) Ball-and-stick representation of the active site structure of W–Cys-type MeFDH1. (*b*) Electron density 2*F*_o_ − *F*_c_ map of W–Cys-type MeFDH1. (*c*) Chemical structure representing the bis­MGD cofactor with the tungsten ion.

The metal ion in the active site of metal-dependent FDHs is coordinated by six ligands in a trigonal prismatic geometry. The rectangular base of the prism is formed by the two di­thiol­ene groups of the bis-MGD (metal-binding pterin guanine dinucleotide cofactor). The other two sites are ligated by the cysteine sulfur (or the seleno­cysteine selenium) and a small ligand, oxygen or sulfur species. In Mo–SeCys-type EcFDH-F, this small ligand was initially assigned as Mo–OH, but it was subsequently reinterpreted as Mo–SH (Raaijmakers & Romão, 2006 ▸). The crystal structure of the W–SeCys-type enzyme from *D. vulgaris* Hildenborough (DvFDH-AB) revealed that an Se atom from a seleno­cysteine residue was bound to the W ion and the sulfur ligand was also determined to be in the oxidized state (Oliveira *et al.*, 2020 ▸). The cryo-EM structure of Mo–Cys-type RcFdsGBACD revealed that the Mo ion was coordinated by six ligands of the bis-MGD cofactor, the cysteine sulfur and a sulfido ligand with similar geometry to the other FDHs. The recent structural analysis of W–Cys-type MeFDH1 in the oxidized state showed, however, that the six ligands coordinated to the W ion were those from the bis-MGD, a cysteine sulfur and a tentative oxygen species based on the coordination bond lengths (Fig. 3 ▸).

As mentioned above, the geometry of the metal coordination sphere is very similar for all W/Mo FDHs. In addition, all FDHs share the common structural feature in the second coordination sphere where the proximal [4Fe–4S] cluster is located next to the pterin ring of the MGD cofactor. A highly conserved lysine residue (K44 in EcFDH-F and K331 in MeFDH1) is located between the pterin cofactor and the proximal [4Fe–4S] cluster. This lysine and the proximal [4Fe–4S] cluster are involved in the electron transfer associated with the catalytic oxidation of the substrate (Raaijmakers & Romão, 2006 ▸). There also exists a pocket region surrounded by strictly conserved histidine and arginine residues near the active site. The putative role of these residues is substrate binding and proton abstraction from the substrate (Hartmann *et al.*, 2016 ▸). A positively charged channel exists in order to transfer the substrate to the buried metal active site. The second channel, which is primarily constructed with hydro­phobic residues, indicates that CO_2_ may be released from the active site through this channel.

## 5. Electrochemical properties

Considering their structural similarity to respiratory chain complex I (NADH ubi­quinone oxidoreductase), FDHs, as well as NAD-reducing hydrogenase, are more primitive enzymes involved in the fundamentals of energy metabolism. Because these enzymes are in the process of evolution, they are not highly specific for electron donors/acceptors that are coupled to the redox reactions of the target substances (CO_2_/formate or H^+^/H_2_). Their immature characteristics from an evolutionary point of view make them suitable for artificial applications, especially incorporation into electrochemical systems.

Bioelectrochemistry is a promising tool for investigating the thermodynamic and kinetic properties of redox enzymes (Mazurenko *et al.*, 2020 ▸; Adachi *et al.*, 2020 ▸; Evans *et al.*, 2019 ▸; Yuan & Minteer, 2019 ▸; Masa & Schuhmann, 2016 ▸). Two strategies can be employed for coupling the enzymatic and electrode reactions. The first strategy is indirect coupling using small redox partners as mediators, referred to as mediated electron transfer (MET)-type bioelectrocatalysis. Several artificial electron donors/acceptors or the natural coenzyme NAD^+^/NADH can be utilized in such systems. MET-type bioelectrocatalysis is very useful for easily evaluating the second-order reaction rate constant between an enzyme and a mediator. In the case of formate oxidation by MeFDH1, the second-order reaction rate constants with several artificial electron acceptors and NAD^+^ displayed characteristic behavior in accordance with a linear free-energy relationship (LFER) (Sakai *et al.*, 2015 ▸). From a scientific point of view, this study showed that MeFDH1 is not specific for NAD^+^, which is the natural electron acceptor. Similar behavior was observed for an NAD-reducing hydrogenase (Shiraiwa *et al.*, 2018 ▸). On the other hand, from an application perspective, these basic data are useful for the design of enzyme electrodes in MET reactions. In the case of CO_2_ reduction, it is desirable to use an artificial mediator with low redox potential, because the redox potential of NAD^+^/NADH is −320 mV, which is less negative than that of CO_2_/formate. Methyl viologen (MV; 1,1′-di­methyl-4,4′-bipyridinium) has been widely used as a common mediator (Parkinson & Weaver, 1984 ▸; Sakai *et al.*, 2015 ▸). In addition, various 2,2′-bipyridinium salt derivatives [1,1′-di­methyl (DM), tri­methyl­ene (TB), ethyl­ene (DB) and tetra­methyl­ene (QB)] are also utilized as suitable mediators for ‘CO_2_ utilization’ with FDHs serving as CO_2_ reductases (Amao *et al.*, 2015 ▸; Amao, 2017 ▸).

**Figure 4:**
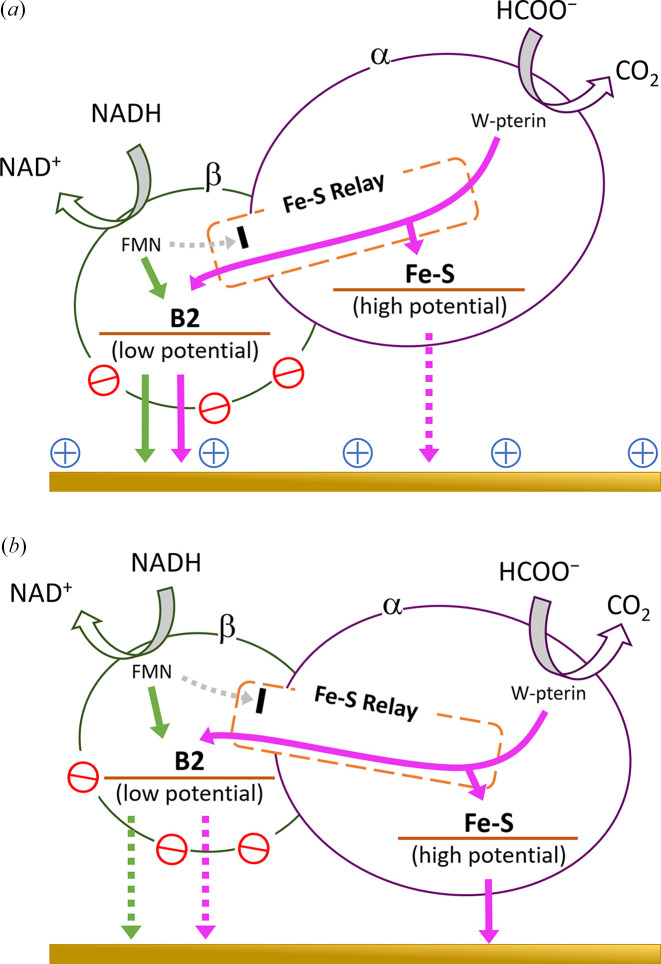
Proposed electron transfer pathways of MeFDH1 on (*a*) 4-ATP/PGE and (*b*) 4-MP/PGE. The green and purple arrows indicate the electron transfer associated with NADH and formate oxidation, respectively. 4-ATP: 4-amino­thio­phenol; 4-MP: 4-mercapto­pyridine; PGE: porous gold electrode. Reproduced from Yoshikawa *et al.* (2022 ▸) with permission from the Royal Society of Chemistry.

The second strategy is direct coupling to the electrode, which is known as direct electron transfer (DET)-type bio­electrocatalysis. To date, six FDHs have been successfully connected to electrodes. Because the long-range electron transfer rate constant decays exponentially with increasing distance, it is necessary to use appropriate electrodes that provide surface characteristics suitable for the fruitful orientation of the enzymes to minimize the distance between the electrode-active site of each enzyme and the electrode surface (Smutok *et al.*, 2022 ▸; Milton & Minteer, 2017 ▸; Takeda & Nakamura, 2021 ▸; Shleev *et al.*, 2005 ▸; Okuda-Shimazaki *et al.*, 2020 ▸; Adachi *et al.*, 2019 ▸). DET-type reactions of FDHs have been reported at the following enzyme-modified electrodes: EcFDH-H-modified graphite–ep­oxy electrodes (Bassegoda *et al.*, 2014 ▸), CnFDH-modified carbon nanotube (CNT)-coated graphite electrodes (Walker *et al.*, 2019 ▸), DvFDH-AB-modified indium tin oxide (ITO)-coated pyrolytic graphite (PG) electrodes (Meneghello, Oliveira *et al.*, 2021 ▸; Miller *et al.*, 2019 ▸), SfFDH-modified bare PG electrodes (Reda *et al.*, 2008 ▸), DdFDH-modified PG electrodes (Cordas *et al.*, 2019 ▸) and MeFDH1-modified mesoporous carbon electrodes (Sakai *et al.*, 2017*b* ▸). So far, Walker and co-workers have been the only researchers to report noncatalytic signals of an FDH with their CnFDH-modified CNT-coated graphite electrodes (Walker *et al.*, 2019 ▸). In the case of MeFDH1, Sakai and co-workers validated the DET-type bioelectrocatalytic interconversion of CO_2_/formate and NAD^+^/NADH. MeFDH1 was not able to directly communicate with the planar electrode, but coating of the electrode with Ketjen Black (KB, a popular mesoporous carbon material) allowed communication between MeFDH1 and the electrode, which was probably attributable to the curvature effect (*i.e.* the average distance between the electrode-active site and electrode surface was reduced by enclosing the enzyme in mesopores of similar diameter to the enzyme). In addition, a gold nanoparticle (AuNP)-embedded KB-coated electrode treated with 4-mercapto­pyridine improved the direct interaction between the enzyme and the electrode, indicating that the pyridine moiety on the AuNPs enhanced the interfacial electron transfer (Sakai *et al.*, 2017*b* ▸). A more detailed study of DET-type bioelectrocatalysis was performed using porous gold electrodes with several surface modifications (Yoshikawa *et al.*, 2022 ▸). Multiple electron transfer pathways in formate oxidation were proposed based on examination of the 3D structure, electrostatic interactions between the electrode and enzyme, and differences between substrates. This study also revealed that there are two electrode-active sites within MeFDH1 (Fig. 4 ▸).

## 6. Catalytic reaction and inhibition mechanism (with a possible substrate transfer system)

Although the catalytic reaction mechanism of formate oxidation by FDHs has been under investigation for several decades, the detailed mechanism remains poorly understood. Numerous reaction mechanisms have been proposed, but it is believed that the reaction mechanism of W-containing FDHs is similar to that of the Mo-containing enzymes. Indeed, in some W-containing enzymes, the W ion can be replaced by Mo (Maia *et al.*, 2015 ▸). In the formate oxidation reaction, two electrons and one proton have to be transferred from the active site to the molecular surface. Thus, electrons transferred to the proximal [4Fe–4S] cluster are subsequently transferred to the other Fe–S clusters in the electron-transfer chain.

**Figure 5:**
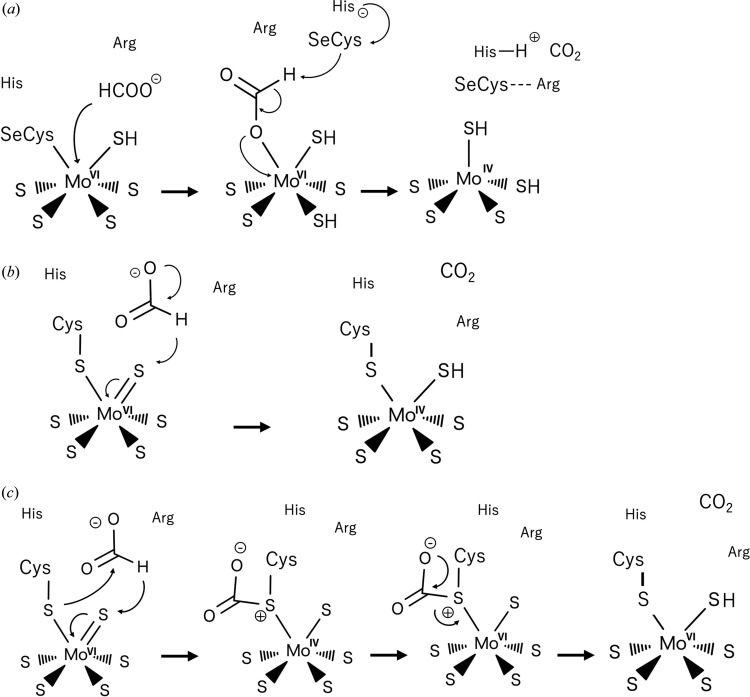
Proposed reaction mechanisms of FDHs. The figures were reproduced and adapted with permission from Stripp *et al.* (2022 ▸).

It has been suggested that the oxidation state of the Mo ion varies between Mo^IV^ and Mo^VI^ during the catalytic reaction mediated by FDHs. Among the proposed mechanisms, the major question is whether the SeCys/Cys and sulfido ligand (or oxygen species) remain coordinated to the metal. Here, we introduce some of the proposed mechanisms. Raaijmakers & Romao (2006 ▸) proposed that the seleno­cysteine residue becomes unbound from the Mo ion upon formate binding to the active site [Fig. 5 ▸(*a*)]. In this mechanism, the seleno­late ion deprotonates the formate and two electrons are transferred to the metal center, resulting in the reduction of Mo^IV^. Subsequently, the proton may be transferred to the histidine (His141 in EcFDH-F) in the binding pocket. In this proposal, the sulfido ligand remains on the metal ion and may play no role in formate oxidation. On the contrary, another proposal by Niks & Hille (2018 ▸) suggested that the formate remains in the substrate-binding pocket and a hydride is abstracted by the sulfido ligand, resulting in SH coordination to the Mo^IV^ ion [Fig. 5 ▸(*b*)]. In this case, the seleno­cysteine residue is not directly involved in the reaction. A very recent time-resolved X-ray crystallography study of W–SeCys-type DvFDH-AB revealed five different structures during enzyme reduction and the presence of a formate molecule in the catalytic pocket (H193, T450, R441 and Q447 in DvFDH-AB) according to the electron density map (Vilela-Alves *et al.*, 2023 ▸). Dong & Ryde (2018 ▸) suggested that the sulfido ligand abstracts a hydride from formate and the carboxyl­ate binds to the cysteine sulfur as an intermediate on the basis of theoretical calculations [Fig. 5 ▸(*c*)].

Most FDHs (*e.g.* EcFDH-F) are known to be inactivated in the presence of O_2_ (Axley *et al.*, 1990 ▸), although some, such as MeFDH1 and those from *Desulfovibrio sp.*, are stable and can be purified under ambient atmosphere without using inhibitors such as azide ions, which are isoelectronic with carbon dioxide. In the case of RcFdsGBACD, it was demonstrated that the sulfido ligand on the Mo ion was replaced by an oxo ligand on exposure to O_2_, suggesting that an azide ion may bind in the catalytic pocket to hinder access of O_2_ to the active site (Duffus *et al.*, 2020 ▸; Schrapers *et al.*, 2015 ▸).

## 7. Potential applications

CO_2_ reduction is a key technology for solving global issues (Min *et al.*, 2020 ▸; Sharma *et al.*, 2020 ▸). Only two types of biocatalysts, namely CO de­hydrogenases (CODHs) and FDHs, are known to directly catalyze CO_2_ reduction (Meneghello, Léger & Fourmond, 2021 ▸). Because these enzymes are able to promote rapid CO_2_ fixation with very small thermodynamic driving forces (low overpotentials), they are expected to be of great help in realizing sustainable energy storage, for which energy efficiency is a fundamental requirement (Meneghello, Léger & Fourmond, 2021 ▸). Some FDHs can also act as CO_2_ reductases and catalyze the reduction of CO_2_ to formate, an interesting and valuable product. Formate is a stable intermediate that can be converted to other C1 compounds such as methanol or methane or used as an industrial feedstock (Glueck *et al.*, 2010 ▸). Furthermore, it can be utilized as a convenient energy source for fuel cells or a non-toxic liquid hydrogen carrier. Table 2 ▸ summarizes recent progress in bioelectrochemical CO_2_ reduction and formate oxidation by FDHs. Although FDH-modified bio-anodes for formate biofuel cells have been studied (Bassegoda *et al.*, 2014 ▸; Reda *et al.*, 2008 ▸; Sakai *et al.*, 2017*a* ▸), we will focus primarily on CO_2_ reduction in this review.

In 2008, Reda *et al.* (2008 ▸) reported the efficient and reversible interconversion of CO_2_ and formate with an SfFDH1-modified graphite electrode, and only low overpotentials were sufficient to reduce CO_2_ to formate with a Faradaic efficiency close to 100%. However, considering the oxygen sensitivity of SfFHD1, it seems advantageous to utilize oxygen-tolerant FDHs. Recently, Yuan *et al.* (2018 ▸) reported the electrochemical properties of an EcFDH-H-functionalized cobaltocene-poly(allyl­amine)-modified glassy carbon electrode. The redox potential of the cobaltocene redox polymer was determined to be −0.576 V, which was more negative than that of CO_2_/HCOO^−^. The redox polymer was able to serve as a suitable redox mediator between EcFDH-H and the electrode for CO_2_ reduction, and the maximum current density of the CO_2_ reduction reached 62 µA cm^−2^. In the case of bio­electrocatalysis with gaseous substrates such as hydrogen, oxygen and CO_2_, the fabrication of gas-diffusion electrode (GDE) systems is one of the best approaches for improving the performance by increasing the substrate diffusion speed (So *et al.*, 2017 ▸). Several studies have demonstrated that metal-dependent FDHs use gaseous CO_2_ as a substrate rather than solvated CO_2_ (carbonate ion species) (Khangulov *et al.*, 1998 ▸; Cooper *et al.*, 1968 ▸; Meneghello, Oliveira *et al.*, 2021 ▸), thus making it reasonable to utilize a GDE system. Sakai *et al.* (2016 ▸) developed an effective gas-diffusion system for CO_2_ reduction using gaseous CO_2_ directly. The authors used MeFDH1 and 1,1′-tri­methyl­ene-2,2′-bipyridinium (TQ) as a redox mediator, which enabled the realization of a very high catalytic current density of 20 mA cm^−2^ for CO_2_ reduction. The incorporation of FDHs into redox hydro­gels has also attracted significant interest in recent decades. The benefits of this strategy include the possibility of increasing the enzyme loading on the electrode, ensuring electrical communication as in conventional mediated electrochemistry in solution, and preventing enzyme leaching from the electrode surface (Heller, 2006 ▸; Gracia & Mecerreyes, 2013 ▸; Ruff, 2017 ▸). This approach has gained in popularity since it was demonstrated that redox hydro­gels could protect oxygen-sensitive enzymes such as hydrogenases against inactivation by O_2_ (Hardt *et al.*, 2021 ▸; Plumeré *et al.*, 2014 ▸; Li *et al.*, 2019 ▸; Fourmond & Léger, 2021 ▸; Cadoux & Milton, 2020 ▸). Szczesny *et al.* (2020 ▸) also constructed a DvFDH-AB-modified GDE, in which carbon cloth was coated with a viologen-based polymer for CO_2_ reduction. By utilizing a low-potential viologen-modified polymer with a redox potential of approximately −390 mV, the GDE exhibited satisfactory stability applicable to a long-term bioelectrocatalytic CO_2_ reduction system. Another redox polymer used for CO_2_ reduction is polyaniline (PANI) hydro­gels, which have been utilized to immobilize ClFDH onto a bioelectrode (Kuk *et al.*, 2019 ▸). This system successfully converted CO_2_ to formate over 12 h of continuous reaction at a high Faradaic efficiency of approximately 93%.

CO_2_ reductases have been studied to find potential applications in CO_2_ capture or provide valuable carbon-based compounds by coupling with photosensitizers to catalyze the photoreduction of CO_2_. The first study of photoelectrochemical CO_2_ reduction catalyzed by an enzyme was reported by Parkinson & Weaver (1984 ▸), who used a semiconductor photoelectrode with PoFDH to catalyze CO_2_ reduction to formate. They used MV as a redox mediator and realized a Faradaic efficiency of approximately 80–93%. Recently, Sokol *et al.* (2018 ▸) coupled a dye-photosensitized photosystem II-based photoanode to a hierarchically structured inverse opal TiO_2_ cathode modified with DvFDH-AB. This system realized light-driven CO_2_ conversion to formate with water as a sacrificial electron donor, at a Faradaic efficiency of around 80%. Besides the use of light energy, coupling CO_2_ reduction with the oxidation of electron sources is an attractive concept for generating formate without an external power supply. Adachi *et al.* (2018 ▸) constructed a bioelectrochemical formate-generation system by combining an MeFDH1-modified GDE with benzyl viologen as a redox mediator and an NiFe hydrogenase-modified GDE. Although a formate-generation rate of 290 pmol cm^−2^ s^−1^ was achieved, the current efficiency was only 20%, which was possibly attributable to electron leakage to dissolved oxygen. Another recent report described a hydrogen-dependent CO_2_ reductase from *A. woodii* (AwFDH2) that was able to use hydrogen as an electron donor for the interconversion of CO_2_ to formate (Schuchmann & Müller, 2013 ▸). AwFDH2 is a native hydrogen-dependent CO_2_ reductase that is a complex of FDH and FeFe hydrogenase. It catalyzes the hydrogenation of CO_2_ to generate formate at a rate of 10 µmol min^−1^ mg^−1^, which is around 1500 times more effective than chemical catalysts (Szczesny *et al.*, 2020 ▸). Furthermore, this study revealed that whole cells of *A. woodii* could be utilized as catalysts to produce formate from CO_2_ and hydrogen, indicating a promising alternative strategy for efficient CO_2_ hydrogenation by employing whole cells as biocatalysts. A number of studies have also demonstrated formate production from CO_2_ by whole-cell biocatalysis using *M. extorquens* AM1 or *Shewanella oneidensis* MR-1 (Hwang *et al.*, 2015 ▸; Jang *et al.*, 2018 ▸; Le *et al.*, 2018 ▸). The formate productivities were reported to be 2.53 and 3.8 m*M* g-cell^−1^ h^−1^ for *M. extorquens* AM1 and *S. oneidensis* MR-1, respectively (Hwang *et al.*, 2015 ▸; Jang *et al.*, 2018 ▸; Le *et al.*, 2018 ▸).

## Figures and Tables

**Table 1:** Metal-dependent FDHs from prokaryotic organisms

Class	Name	Organism	Metal	Fifth ligand	Reference
Methyl­otrophs	MeFDH1†	*Methyl­orubrum extorquens* AM1‡	W	Cys	(Laukel *et al.*, 2003 ▸)
	MeFDH2	*Methyl­orubrum extorquens* AM1	Unknown	Unknown	(Chistoserdova *et al.*, 2004 ▸)
	MeFDH3	*Methyl­orubrum extorquens* AM1	Unknown	Unknown	(Chistoserdova *et al.*, 2004 ▸)
	MeFDH4	*Methyl­orubrum extorquens* AM1	Unknown	Unknown	(Chistoserdova *et al.*, 2007 ▸)
	MspFDH	*Methyl­obacterium sp.* RXM	Mo	Cys	(Duarte *et al.*, 1997 ▸)
	MtFDH	*Methyl­osinus trichosporium*	Mo	Cys	(Jollie & Lipscomb, 1991 ▸)
Methano­gens	MfFDH	*Methano­bacterium formicicum*	Mo	Cys	(Godfrey *et al.*, 1987 ▸)
	MmFDH1	*Methano­coccus maripaludis*	Unknown	Unknown	(Wood *et al.*, 2003 ▸)
	MmFDH2	*Methano­coccus maripaludis*	Unknown	Unknown	(Wood *et al.*, 2003 ▸)
	MvFDH1	*Methano­coccus vannielii*	Mo	Cys	(Jones & Stadtman, 1981 ▸)
	MvFDH2	*Methano­coccus vannielii*	W	SeCys	(Jones & Stadtman, 1981 ▸)
Sulfate-reducing bacteria	DaFDH	*Desulfovibrio alaskensis*	W	SeCys	(Brondino *et al.*, 2004 ▸)
	DdFDH	*Desulfovibrio desulfuricans*	Mo	SeCys	(Costa *et al.*, 1997 ▸)
	DgFDH	*Desulfovibrio gigas*	W	SeCys	(Almendra *et al.*, 1999 ▸)
	DvFDH2	*Desulfovibrio vulgaris*	Mo	Cys	(Sebban *et al.*, 1995 ▸)
	DvFDH-AB†	*Desulfovibrio vulgaris*	W	SeCys	(Oliveira *et al.*, 2020 ▸)
	SfFDH1	*Syntrophobacter fumaroxidans*	W	SeCys	(de Bok *et al.*, 2003 ▸)
	SfFDH2	*Syntrophobacter fumaroxidans*	W	SeCys	(de Bok *et al.*, 2003 ▸)
Acetogens	CaFDH2	*Clostridium acidurici*	W	SeCys	(Wang *et al.*, 2013 ▸)
	CcFDH	*Clostridium carboxidivorans*	W	SeCys	(Alissandratos *et al.*, 2013 ▸)
	CfFDH	*Clostridium formico­aceticum*	W	SeCys	(Leonhardt & Andreesen, 1977 ▸)
	ClFDH	*Clostridium ljundahlii*	W	Unknown	(Kuk *et al.*, 2019 ▸)
	CpFDH	*Clostridium pasteurianum*	Mo	Cys	(Liu & Mortenson, 1984 ▸)
	CtFDH	*Clostridium themo­aceticum*	W	SeCys	(Ljungdahl & Andreesen, 1978 ▸)
	AwFDH2	*Acetobacterium woodii*	Mo	Unknown	(Schuchmann & Müller, 2013 ▸)
	TkFDH	*Thermoanaerobacter kuvui*	W	Unknown	(Schwarz *et al.*, 2018 ▸)
Alphaproteobacteria	RpFDH	*Rhodopseudomonas palustris*	Unknown	Unknown	(Larimer *et al.*, 2004 ▸)
	RaFDH†	*Rhodobacter aestuarii*	Mo	Unknown	(Min *et al.*, 2020 ▸)
Betaproteobacteria	CnFDH	*Cupriavidus necator* §	Mo	Cys	(Yu *et al.*, 2017 ▸; Oh & Bowien, 1998 ▸; Friedebold & Bowien, 1993 ▸)
	RcFDH†	*Rhodobacter capsulatus*	Mo	Cys	(Hartmann & Leimkühler, 2013 ▸)
Gammaproteobacteria	EcFDH-F	*Escherichia coli*	Mo	SeCys	(Raaijmakers & Romão, 2006 ▸)
	EcFDH-H	*Escherichia coli*	Mo	SeCys	(Axley *et al.*, 1990 ▸)
	EcFDH-N	*Escherichia coli*	Mo	SeCys	(Jormakka *et al.*, 2002 ▸)
	EcFDH-O	*Escherichia coli*	Mo	SeCys	(Benoit *et al.*, 1998 ▸)
	PaFDH	*Pseudomonas aeruginosa*	Mo	SeCys	(Godfrey *et al.*, 1987 ▸)
	PoFHD	*Pseudomonas oxalaticus*	Unknown	Unknown	(Parkinson & Weaver, 1984 ▸)
Campylobacteria	WsFDH	*Wolinella succinogenes*	Mo	Cys	(Lenger *et al.*, 1997 ▸)
	SmFDH	*Sulfurospirillum multivorans*	Unknown	Unknown	(Schmitz & Diekert, 2003 ▸)
Actinomycetia	CgFDH	*Corynebacterium glutamicum*	Mo	Cys	(Witthoff *et al.*, 2012 ▸)

† These enzymes have been reported to be oxygen tolerant.

‡ Previously known as *Methyrobacterium extorquens* AM1.

§ Previously known as *Ralstonia eutropha* or *Alcaligenes eutrophus*.

**Table 2:** Recent progress in electrochemical CO_2_ reduction and formate oxidation by FDHs

	Current density (µA cm^−2^)			
FDHs	CO_2_ reduction	Formate oxidation	Electrode configuration and reaction conditions	Reference
SfFDH1	80	–	Graphite-ep­oxy electrode −800 mV versus Ag|AgCl/pH 5.9/37°C	(Reda *et al.*, 2008 ▸)
	–	180	Graphite-ep­oxy electrode 200 mV versus Ag|AgCl/pH 7.8/37°C	(Reda *et al.*, 2008 ▸)
EcFDH-F	62	–	Cobaltcene-poly (allyl­amine)-modified glassy carbon electrode −660 mV versus SHE/pH 6.0	(Yuan *et al.*, 2018 ▸)
MeFDH1	20000	–	KB-modified GDE with TQ† −800 mV versus Ag|AgCl/pH 6.5/30°C	(Sakai *et al.*, 2016 ▸)
	–	30000	Viologen-functionalized polymer and KB-modified carbon cloth −300 mV versus Ag|AgCl/pH 7.0/40°C	(Sakai *et al.*, 2017*a* ▸)
DvFDH-AB	533		Viologen polymer-modified GDE −600 mV versus SHE/pH 6.0	(Szczesny *et al.*, 2020 ▸)

† 1,1′-Tri­methyl­ene-2,2′-bipyridinium dibromide (TQ) was added to the solution.
